# Experiences in Nature and Environmental Attitudes and Behaviors: Setting the Ground for Future Research

**DOI:** 10.3389/fpsyg.2019.00763

**Published:** 2019-04-09

**Authors:** Claudio D. Rosa, Silvia Collado

**Affiliations:** ^1^Department of Development and Environment, Universidade Estadual de Santa Cruz, Ilhéus, Brazil; ^2^Department of Psychology and Sociology, University of Zaragoza, Teruel, Spain

**Keywords:** biophilia, connection to nature, ecological behavior, environmental identity, experiences of nature, nature-based recreation, nature exposure, outdoor recreation

## Abstract

There is empirical evidence suggesting a positive link between direct experiences in nature and people’s environmental attitudes (EA) and behaviors (EB). This has led researchers to encourage more frequent contact with nature, especially during childhood, as a way of increasing pro-environmentalism (i.e., pro-EA and pro-EB). However, the association between experiences in nature and EA/EB is complex, and specific guidelines for people’s everyday contact with nature cannot be provided. This article offers an overview of the research conducted until know about the relation between experiences in nature and pro-environmentalism, and opens up new inquiries for future research. We begin with an introduction to people’s current tendency toward an alienation from the natural world and set out the objectives of the article. It is followed by three main sections. The first one reports on what experiences in nature refer to, how and where they occur. The second section describes the different approaches used to investigate and interpret the experiences in nature-EA and EB relation. The last section provides suggestions for future research. We close by making some final remarks about the importance of (re)stablishing a greater interaction with nature for people’s pro-EA and EB.

## Introduction

Experiences in nature are associated with several benefits, such as recovery of cognitive resources (Hartig et al., 2014), increased pro-environmental attitudes (EA) (Chawla and Derr, 2012) and behaviors (EB) (Evans et al., 2018), more frequent physical activity (Schaefer et al., 2014) and increases pro-social orientation (Joye and Bolderdijk, 2014). In spite of these positive effects of people’s contact with nature, there is mounting evidence indicating that people’s direct contact with nature is diminishing (Zaradic et al., 2009; Soga and Gaston, 2016). Several reasons have been suggested for this growing alienation from the natural world, including increased urbanization rates, more frequent use of new technologies for entertainment, and the perception of nearby natural places as insecure (Clements, 2004; Tandon et al., 2012; Soga and Gaston, 2016; Larson et al., 2018a). Researchers have warned that this lack of experiences in nature may have negative consequences for people’s pro-environmentalism (i.e., their pro-EA and pro-EB) (Soga and Gaston, 2016; Evans et al., 2018; Rosa et al., 2018), which could lead to detrimental consequences for the environment (Evans, 2019). As a result, there has been a proliferation of initiatives (e.g., no child left inside) and publications (e.g., Louv, 2008) targeted at the general public with the aim of encouraging a more frequent contact with nature from early childhood. Yet, is the link between experiences in nature and pro-environmentalism as well-stabilished as the studies above suggest? Based on what is currently known about experiences in nature and pro-environmentalism, the main goal of this article is to outline a number of issues for future research on this area. To do this, we first review and synthetize the different approaches from which the relation between experiences in nature and pro-environmentalism has been studied. In our review, EA are seen as a “collection of beliefs, affect, and behavioral intentions a person holds regarding environmentally related activities or issues" (Schultz et al., 2005, 458). This definition is quite broad and, as such, we consider in our review studies evaluating people’s ecological beliefs (Van Liere and Noe, 1981), connectedness to nature (Mayer and Frantz, 2004), place attachment (Hou et al., 2005), biophilia (Zhang et al., 2014), and willingness to engage on EB (Larson et al., 2018b).

Experiences in nature are positively associated with stronger pro-environmentalism, such as emotional affinity toward nature (Kals et al., 1999), willingness to conserve biodiversity (Soga et al., 2016), willingness to pay for the conservation of urban green spaces (Lo and Jim, 2010), and pro-EB (Evans et al., 2018). Overall, researchers have found a positive link between EA and EB (Bamberg and Möser, 2007). This is in line with the Theory of Planned Behavior (Ajzen, 2011), and the Value-Belief-Norm-Theory (Stern et al., 1999), and have been supported empirically (e.g., Dunlap et al., 2000; Markle, 2013). As a general trend, the relation between experiences in nature and EB is mediated by EA (Larson et al., 2011; Pensini et al., 2016; Otto and Pensini, 2017; Rosa et al., 2018). Several ideas have been offered to explain why experiences in nature positively influence EB. These explanations include increased biocentric values (Larson et al., 2011), connectedness to nature (Otto and Pensini, 2017), and beliefs about the New Environmental Paradigm (Collado et al., 2013), a stronger sense of place attachment (Lawrence, 2012), increased positive emotions (Mayer et al., 2009) and renewal of depleted attentional capabilities (i.e., psychological restoration) (Byrka et al., 2010; Collado and Corraliza, 2015; Wyles et al., 2017), and a stronger sense of morality toward the environment (Hahn and Garrett, 2017).

In spite of the positive associations between experiences in nature and pro-environmentalism reported in previous studies, this relation is a complex one (Clayton et al., 2017). This complexity has, to our knowledge, been overlooked. Our departure point is an overview of the research findings in this area, distinguishing six different approaches used to examine the relation between people’s experiences in nature and pro-environmentalism. Then, we address what we consider to be more urgent in terms of future research. The article has been organized in three main sections describing: (a) experiences in nature: how and where they occur, (b) approaches used to investigate and interpret the link between experiences in nature and pro-environmentalism, and (c) future research.

## Experiences in Nature: How and Where They Occur

By experiences in nature we refer to time spent in natural areas, including wild natural areas, such as forests, but also nearby natural environments, like urban parks, gardens, and vacant lots (Chawla and Derr, 2012; Keniger et al., 2013; Rupprecht et al., 2016). Several authors have defined experiences in nature by focusing on specific aspects of the person–nature interaction. For instance, Keniger et al. (2013) considered the motivation for people’s interactions with nature (i.e., intentional or non-intentional). According to these authors, there are two different types of experiences in nature. First, individuals can have incidental contact with nature (i.e., experiencing nature as a by-product of another activity, such as walking the dog). Second, experiences in nature can be direct and intentional, when people have the intention to be in direct contact with nature. Direct and intentional contact with nature in an esthetically pleasing environment is thought to be the best way for people to connect with nature, as it involves the use of diverse senses (Lumber et al., 2017; Giusti et al., 2018). In line with this, Mayer et al. (2009) found a higher increase in connectedness to nature after people had a direct experience in nature compared to an indirect contact with nature through a video.

Another way of defining people’s experiences in nature is by focusing on the type of activity conducted in the natural setting [see Berns and Simpson (2009) for a review]. The authors distinguish three types of experiences in nature: consumptive, mechanized, and appreciative. According to Berns and Simpson (2009), consumptive activities in nature refer to taking something from the environment for your own use (e.g., fishing, hunting). In turn, mechanized activities in nature are activities in which mechanized equipment is used to interact with nature (e.g., off road vehicles). Last, appreciative activities in nature relate to enjoying the natural environment (almost) without altering it through self-propelled non-mechanized activities (e.g., surfing, birdwatching, hiking). Research evidence suggests that appreciative experiences in nature are the ones more strongly linked to pro-environmentalism.

Clayton et al. (2017) went a step further in defining people’s experiences in nature. The authors considered the social context in which experiences in nature take place. In their view, experiences in nature can be self-directed, when the person interacts freely with the environment or other-directed, when the person interacts with the natural environment following someone else’s guidance (e.g., tour guide). In addition, interactions with nature can be solitary experiences or they can happen in the company of others and, at the same time, they can provide positive or negative emotional responses (Clayton et al., 2017).

## Approaches Used to Investigate and Interpret the Link Between Experiences in Nature and Pro-Environmentalism

According to our review of the research findings in this area, we can distinguish six main research approaches to examine the relation between experiences in nature and pro-environmentalism. Below, we describe the main findings derived from each of these approaches. These are summarized in Table 1.

**Table 1:** Description of the approaches applied to the study of the relation between experiences in nature and EA and EB.

Approach	Brief description	Main insights	Example of classical studies
1. Significant life experience (SLE)	Studies analyzing the reasons why environmental activists devoted their lives to taking care of the environment. Childhood experiences in nature have been identified as a main driver for adulthood pro-environmentalism.	SLE literature shows that it is important to consider lifetime experiences with nature in order to understand current EA and EB.	Tanner, 1980
2. Comparison between nature-based recreationists EA and EB	Studies analyzing if individuals involved in different nature-based recreational activities have distinct EA and EB. For example, comparing EA and EB of hunters with those of birdwatchers.	These studies indicate that the type of interaction with nature (e.g., consumptive vs. appreciative) needs to be considered when analyzing the link between interactions with nature and pro-environmentalism.	Dunlap and Hefferman, 1975
3. Specialization	Studies analyzing whether differences in pro-environmentalism are linked to nature-based recreationist specialization (e.g., experience and technical skills a person has on a recreational activity).	This literature provides insights about the relevance of individuals’ specialization on a nature-based recreational activity to the understanding of their EA and EB.	Bryan, 1977
4. Interactions with nature influences pro-environmentalism	Studies analyzing whether interactions with nature can increase people’s pro-environmentalism.	Positive direct experiences in nature are linked to an increase in pro-environmentalism.	Mayer et al., 2009
5. EA influence interactions with nature	Studies analyzing whether people’s EA can influence their pattern of interactions with nature.	This literature suggests that EA may be a driver for interactions with nature.	Lin et al., 2014
6. The perceived benefits of interactions with nature as predictors of pro-environmentalism	Studies analyzing if the perceived benefits of interactions with nature (e.g., restoration, pleasure) are associated with people’s pro-environmentalism.	Studies on this approach suggest that people EA and EB may change when they realize the benefits of nature to their lives.	Hartig et al., 2001

First, the relation between people’s experiences in nature and pro-environmentalism has been examined from research in the area of significant life experience (SLE). According to SLE studies, childhood positive experiences in nature are the main factor predicting pro-environmentalism later in life (Tanner, 1980; Chawla, 1999; Corcoran, 1999; Chawla and Derr, 2012). These studies are mainly qualitative and retrospective. For instance, Tanner (1980) evaluated the experiences that 45 environmental activists recalled as being more important for their decision of working as environmental conservationists. According to Tanner’s (1980) findings, experiences in nature as a child were the main predictor of their choice. Recently, Cagle (2018) conducted a retrospective investigation about experiences in nature over time. Participants were 12 environmentally committed faculty members of Duke University (United States). Her results supported previous findings, indicating that childhood experiences in nature were important for the formation of a bond with nature that lasts until adulthood. In line with these results, Gray and Pigott (2018) found that people who participated in a 2-year nature-immersive activity when they were about 15 years old recalled that experience, 30 years later, as a motivating factor to choose a career related to conservation, wilderness guiding, and environmental education.

A second research approach involves the evaluation of the effect that different types of activities in nature may have in pro-environmentalism. For instance, Knopp and Tyger (1973) found that individuals engaged in an appreciative activity (ski touring) held stronger EA than those involved in a mechanized activity (snowmobiling). Similarly, Wolsko and Lindberg (2013) found a positive link between participating in appreciative activities and connectedness to nature, and a negative association between participating in motorized activities and connectedness to nature. Due the cross-sectional design of these studies, it is not possible to establish whether individuals with strong EA prefer appreciative activities as argued by Bjerke et al. (2006), or if participating in appreciative activities improves individuals’ EA, as argued by Dunlap and Hefferman (1975). Yet, both perspectives could be correct (Jackson, 1986). In concordance with Dunlap and Hefferman’s (1975) perspective, Wyles et al. (2017) conducted an experimental study analyzing the effect of three appreciative activities (e.g., coastal walking) in individuals’ intention to engage on responsible environmental behaviors and found that the three activities increased participants’ intention to engage in these behaviors in a similar way.

A third line of study consists on analyzing the possible differences on nature-based recreationists’ EA and EB based on their specialization on a specific nature-based activity. Specialization is generally considered a multidimensional concept formed by behavioral, affective, and cognitive factors (Scott and Shafer, 2001; Garlock and Lorenzen, 2017; Kim and Song, 2017). It is commonly assessed by a series of factors such as recreationists’ experience in a nature-based activity, the importance of this activity for the individual’s lifestyle, the technical skills required by this specific activity, and the expenses of the activity (McFarlane and Boxall, 1996; Scott and Shafer, 2001; Garlock and Lorenzen, 2017; Kim and Song, 2017). In comparison to less specialized recreationists, the more specialized ones are expected to be more experienced, to show greater mastery of the techniques associated with the leisure activity, to spend more money on the activity, and to perceived the activity as more relevant for his/her lifestyle (Scott and Shafer, 2001). Previous studies have investigated if more specialized nature-based recreationists such as birdwatchers (McFarlane and Boxall, 1996), anglers (Garlock and Lorenzen, 2017), and boaters (Jett et al., 2009) hold distinct EA and EB than less specialized nature-based recreationists. One hypothesis is that as a person specializes in an activity, pro-EA and engagement in pro-EB can increase (Bryan, 1977; McFarlane and Boxall, 1996). This may occur because more specialized recreationists perceive the recreational activity they practice as more relevant for them than less specialized recreationists, and these activities depend upon natural resources (Bryan, 1977). Hence, more specialized recreationists may hold greater concern for the maintenance of the natural resources where the activity is conducted than less specialized recreationists. Literature findings generally support this hypothesis, with more specialized individuals reporting higher levels of environmental concern and greater engagement in pro-environmental behaviors (Thapa et al., 2006).

A fourth approach in the study of people’s experiences in nature and pro-environmentalism involves the prediction that direct experiences in natural environments foster pro-EA and EB. In the last years, several interventions have showed that experiences in nature can foster children’s (Crawford et al., 2017; Schneider and Schaal, 2017) and adults’ connectedness to nature (Lumber et al., 2017; Richardson and McEwan, 2018), an important predictor of pro-EB (Tam, 2013; Frantz and Mayer, 2014). For example, Barton et al. (2016) found an increase in adolescents’ connectedness to nature after a wildness expedition. Evans et al. (2018) conducted a longitudinal study in which they found, after controlling for possible confounding variables (e.g., child environmental behavior), that one of the main predictors of young adults’ EB was time spent outdoors during childhood. Considering these results, it does not come as a surprise that direct contact with nature has been seen as a predictor of pro-environmentalism in the environmental education domain, with several environmental education projects focusing on nature experiences as a way of promoting EA and EB (Evans et al., 2007; Duerden and Witt, 2010). For example, De Dominicis et al. (2017) found an increase on place attachment, pro-EA, and self-reported pro-EB after a nature-based environmental education program. Similarly, Collado et al. (2013) found that time spent in nature-based summer camps, with and without environmental education within their daily program, had positive effects on EA and EB.

The fifth approach in the study of the link between experiences in nature and pro-environmentalism relates to the influence of EA on the way people interact with nature. Empirical evidence supports that EA motivate people to interact with nature (Lin et al., 2014; Soga and Gaston, 2016; Lin et al., 2017) and influences their choice for nature-based activities (Bjerke et al., 2006; Thapa, 2010; Marques et al., 2017). For example, Lin et al. (2014) found that people who felt more connected to nature were more likely to visit parks and to spend more time on their private yard than people who felt less connected to nature. Similarly, Bjerke et al. (2006) noted that people’s environmental beliefs were associated to their preference for nature-based activities. They found that individuals with stronger environmental beliefs reported higher preference for activities, like scenery photographing and mountaineering, compared to individuals with weaker environmental beliefs.

The last approach involves the perceived benefits of experiences in nature and how these perceptions lead to pro-environmentalism (Hartig et al., 2007; Byrka et al., 2010; Lee, 2011; Collado and Corraliza, 2015; Lee and Jan, 2018; Whitburn et al., 2018). Natural settings are often perceived as places linked to wellbeing (Carrus et al., 2015), where attentional resources can be recovered (Carrus et al., 2017) and energy can be regained (Ryan et al., 2010). For instance, Lee (2011) reported that individuals’ satisfactions with time spent in nature was related to their conservation commitment and pro-EB. Similarly, Collado and Corraliza (2015) found that children who reported higher restoration after spending time in nature held stronger pro-EA which, in turn, led to conducting pro-EB more frequently. Hartig et al. (2001, 2007) reached similar conclusions with adult samples. These results suggest that the link between experiences in nature and pro-environmentalism can be explained, at least partly, by the benefits people perceive they obtain from time spent in nature.

Overall, the six approaches described suggest that there is a positive relation between experiences in nature and pro-environmentalism. However, as previously indicated, this relation is a complex one (Clayton et al., 2017) and contradictory results have been found in the literature. For example, considering SLE, Howell and Allen (2016) concluded that nature experiences during childhood did not have a major formative influence on 85 people involved in climate change education and mitigation. Their findings suggest that for their participants, factors different from nature experiences during childhood, such as altruistic concerns about climate change, may play a major role in the way they behave toward the environment during adulthood. Also, one would expect that spending time in natural areas while being part of an EE program provides an additional benefit to pro-environmentalism due to the relevant formal environmental information that participants received through EE programs (Kuo et al., 2019). However, this is not always the case. In fact, EE programs could constrain individuals’ willingness to experience nature freely, and the provision of environmental knowledge may be seen as boring (Duerden and Witt, 2010; Collado et al., 2013). Thus, environmental educators have made an effort to develop ways of learning in nature while keeping the experience as fun as possible (Crawford et al., 2017; Schaal et al., 2018).

Contradictory results were also found when considering how different types of experiences in nature were associated to EB (Cooper et al., 2015). The authors expected appreciative activities to be more strongly associated to pro-EB than consumptive activities. However, they found that individuals participating regularly in both hunting and birdwatching (hunter–birdwatcher) were more likely to engage in specific conservation behaviors than individuals participating regularly either in hunting (consumptive) or birdwatching (appreciative). Regarding specialization, Jett et al. (2009) found a negative association between the level of specialization of boaters and their attitudes related to marine conservation. According to their results, more specialized individuals were less likely to agree that, regardless of regulations, manatees deserve to be protected and that reducing vessel speed is an effective strategy for marine conservation. Jett et al. (2009) believe that the reason behind these results is the aversion of more specialized individuals to limitations to their leisure pursuit. The benefits people attribute to time spent in nature (e.g., psychological restoration, well-being) can be moderated by people’s EA (Davis and Gatersleben, 2013; Knez and Eliasson, 2017; Craig et al., 2018). In line with this idea, Knez and Eliasson (2017) found that people feel greater well-being when visiting outdoor settings if they held stronger attachment to these settings. Likewise, Craig et al. (2018) noted that people more connected to nature perceive nature experiences as more pleasurable than those whose connection to nature is lower.

To sum up what we can learn from these approaches, experiences in nature during childhood and adulthood are positively linked to pro-environmentalism (Tanner, 1980; Crawford et al., 2017; Lumber et al., 2017; Rosa et al., 2018). The effect of nature experiences on people’s EA and EB depends on several factors, such as type of experience in nature (Knopp and Tyger, 1973), perceived benefits of these experiences (Hartig et al., 2001; Carrus et al., 2017), and the person’s level of specialization in a certain activity (Bryan, 1977). However, the results found until now are mixed, indicating that the relation between experiences in nature, EA, and EB is not simple, and that there is a need for more nuanced research on the topic. Following, we suggest what we consider to be the most urgent lines of research.

## Future Research

By now we have reviewed the diverse approaches used to investigate and interpret the relation between experiences in nature and EA and EB. We acknowledge the scientific advances done on this topic during the last four decades. Yet, the relation between experiences in nature and pro-environmentalism is, in our opinion, not completely understood. There are some knowledge gaps, contradictory results, and methodological issues that preclude us to firmly claim how and when experiences in nature lead to an increase in pro-environmentalism. Below, we highlight six areas for further investigation.

First, researchers should acknowledge the possibility of a cyclical relation between experiences in nature and pro-environmentalism. There is theoretical and empirical support for two perspectives: (1) experiences in nature enhance EA, leading to EB; and (2) EA can influence people’s patterns of interaction with nature. Whereas several experiments have supported the first perspective (Mayer et al., 2009; Crawford et al., 2017; Lumber et al., 2017), the second one has not been, to our knowledge, experimentally proven. The experimental examination of the second perspective as well as of the possible cyclical relation between experiences in nature and pro-environmentalism awaits future research.

Second, a clear definition of what researchers understand by experiences in nature is still missing. Experiences in nature can have many different forms. For instance, they can be direct and incidental (e.g., experiencing nature while walking the dog), direct and intentional (e.g., surfing), alone or with company (e.g., family, friends), and during work or leisure time. Moreover, even if a definition is given (e.g., Collado and Sorrel, 2019), it is generally broad, and focused on contact with green natural elements. The possible effects that exposure to different types of natural environments have in pro-environmentalism, including water bodies (e.g., oceans, lakes, rivers), settings with different geological and orographic characteristics (e.g., mountain area versus a plain natural setting), and different meteorological conditions remain underexplored. For instance, Talebpour (2018) found that fifth-grade students who participated in a nature immersive program during inclement weather had a decrease in their connectedness to nature compared to before the immersion, whereas students who had the same nature experience with better weather conditions had an increase in connectedness to nature after the immersive program. Future studies should describe in detail how experiences in nature take place, including the meteorological conditions as well as the natural elements participants might encounter. The possible positive impact of direct and visual contact with non-human animals on people’s pro-environmentalism also needs further consideration [see Young et al. (2018) for a review]. Finally, the word *nature* can have different meanings for people from different cultures (Wohlwill, 1983; Collado et al., 2016; Profice, 2018) and as such the definition of experiences in nature should take into account participants’ cultural background. For instance, some people may consider humans are part of nature while others may not (Wohlwill, 1983; Mayer and Frantz, 2004) and differences in the esthetic perception of nature have consequences for biodiversity protection (Williams and Cary, 2002). Given that the meaning of nature is influenced by our social context (Wohlwill, 1983), researchers should pay special attention to people’s conceptions of nature in cross-cultural research.

Third, a clear definition of experiences in nature should be accompanied by a valid measure of frequency of contact with nature. This would facilitate the comparison of results among studies as well as the generalization of findings. Whereas there are likely hundreds of published studies on the topic, to our knowledge, a validated and reliable measure of frequency of contact with nature has not been developed. Researchers generally develop or adapt *ad hoc* measures for their own studies. Experts on the appraisal of measures have argued that the use of non-validated and reliable measures may be a waste of resources and unethical (Mokkink et al., 2018). In light of the research lines described above, researchers may need to think of different measures for different population groups (e.g., children and adults), as well as to be used in different contexts in which experiences in nature are likely to differ (e.g., developed versus developing countries).

Forth, the type of activities in nature leading to pro-environmentalism are fairly unknown, as well as the social context in which these activities occur. We know from previous studies that positive, appreciative experiences in nature may play a stronger role in the formation of pro-environmentalism than consumptive and mechanized activities but we know little about the different effect that different appreciative activities may have (Wyles et al., 2017). For instance, would going for a walk in nature have the same effect as sitting and relaxing in a park? In line with this idea, Giusti et al. (2018) developed a framework to understand how children connect with nature. Their results showed that some forms of experiencing nature (those that engaged children’s senses were children-driven and thought provoking) were more effective to connect children with nature than others (e.g., structured activities). And, would the link between experiences in nature and pro-environmentalism be stronger when these experiences take place alone or when they take place with the company of close ones? Retrospective studies have shown that adults recall positive experiences in nature during childhood in the company of others as the main driver of their current pro-environmentalism (Chawla and Derr, 2012). Yet, when in need of psychological restoration, adults prefer to spend time alone in a natural environment (Staats and Hartig, 2004). Whether there is a difference between spending time in nature alone or with friends in terms of pro-environmentalism and the possible effect of the foreseeable change of children’s social group as they grow up deserves further exploration. Future longitudinal research should focus on how experiences in nature change through the lifespan (Cagle, 2018) and how these different interactions in the natural environment lead to pro-environmentalism.

Fifth, participants’ sociodemographic characteristics should be considered in the experiences in nature–pro-environmentalism relation. These include age, gender, education, place of residence (urban vs. rural), and political ideology [see Gifford and Nilsson (2014) for a review]. Younger people, women, and liberals tend to be more open to improve their EA than older people, men, and conservatives (Heberlein, 2012; Gifford and Nilsson, 2014). Thus, experiences in nature may have a greater impact on young women with liberal ideas. Given that individuals with a lower educational level generally hold weaker EA (Gifford and Nilsson, 2014), they may benefit the most from experiences in nature. Also, people living in rural areas tend to have more frequent contact with nature (Hinds and Sparks, 2008; Muslim et al., 2017) than those living in cities. Rural residents also differ from urban ones in their preference for different types of landscapes (Williams and Cary, 2002) and their daily type of contact with nature (Collado et al., 2015) which can influence the outcomes of experiences in nature. For example, Bixler et al. (1994) found that urban residents tend to express fear and discomfort when spending time in wildland areas. Williams and Cary (2002) found that urban residents held a stronger preference for more grazed woodland landscapes than rural residents do. Considering children, Collado et al. (2015) concluded that children living in mountain rural areas experienced nature more freely than those living in cities. Even though these studies suggest that the effects of experiences in nature on people’s pro-environmentalism may be moderated by sociodemographic characteristics, only a few studies have included them in their studies (e.g., Mayer et al., 2009; Larson et al., 2011; Collado et al., 2015; Rosa and Profice, 2018). These individual factors should be considered in future research about the experiences in nature–pro-environmentalism link.

Sixth, most of the studies revised through this paper are cross-sectional and their samples non-representative. This limits causal inferences and the generalization of the results to larger populations. Also, most of the studies investigating EB and experiences in nature assessed self-report (vs. observed) behaviors. This could be an issue because self-reports can be influenced by diverse biases such as social desirability, mood state, and consistency motif (Podsakoff et al., 2003). Hence, self-reported behaviors may not correspond to observable behaviors (Kormos and Gifford, 2014). Moreover, most of studies investigating the association between childhood experiences in nature and adults’ pro-environmentalism relied solely on adults’ self-reported experiences in nature during childhood (e.g., Wells and Lekies, 2006; Larson et al., 2011; Pensini et al., 2016; Asah et al., 2018; Rosa et al., 2018) with few exceptions (Liddicoat and Krasny, 2014; Evans et al., 2018; Gray and Pigott, 2018). Adults’ self-reports of childhood experiences in nature could be imprecise and may be influenced by their actual EA (Kals et al., 1999; Wells and Lekies, 2006). Therefore, there is a need for representative surveys, more reliable measures of EB and experiences in nature, and longitudinal studies estimating experiences in nature and EA and EB at different time-points.

## Final Remarks

There is plenty of evidence supporting the positive link between experiences in nature, EA, and EB. We agree with previous researchers encouraging a more frequent exposure to nature both for adults and children, as this can have positive effects both on pro-environmentalism (Crawford et al., 2017; Lumber et al., 2017) and health (Hartig et al., 2014; Kuo et al., 2019). However, the complexity of the positive association between experiences in nature and pro-environmentalism urges future studies with which researchers can provide plausible explanations of this positive link, as well as offer more specific guidelines for translating empirical studies into every day practices. For example, future studies can help to shed some light about which are the best type experiences in nature to improve pro-environmentalism (e.g., Giusti et al., 2018), and ways to increase people’s contact with nature within their daily life (e.g., Soga et al., 2018).

## Author Contributions

CR and SC conceived the ideas included in the manuscript and wrote the manuscript.

## Conflict of Interest Statement

The authors declare that the research was conducted in the absence of any commercial or financial relationships that could be construed as a potential conflict of interest.
